# Dr. Marcet's Essay on Calculous Disorders

**Published:** 1818-06

**Authors:** 


### *Dr.* Marcet's *Essay on Calculous Disorders.*

( Continued from page 406. )

### Chap. 6. *On the Analysis of Urinary Calculi, with a View to their easy Discrimination.*

Under this head the author observes, that a blow-pipe, a candle, and a small pair of tongs to hold a particle of the calculus while under examination, are in most cases the only apparatus required to determine the prevailing nature of urinary concretion.

The brownish coloured, compact, rather hard and smooth flattened oval calculus, is generally lithic. To ascertain it, a particle is exposed to the action of flame directed by the blow-pipe; when, if lithic acid prevails, the fragment blackens, emits a smoke having a strong odour, and is gradually consumed, leaving a minute quantity of white ash, which is usually alkaline.

The presence of lithic acid may also be determined by scraping off a little of the calculus into a watch glass, pouring upon it a few drops of caustic potash, and exposing it to heat. The lithic acid is thus dissolved, leaving a residue dependant on the composition of the calculus. By adding any acid to this solution, a white precipitate of pure lithic acid is formed.

Another mode is that of dissolving a particle of the calculus in nitric acid, and applying heat, when the lithic acid disappears; and if the solution be evaporated to dryness, the residue assumes a beautiful pink colour.

Phosphat of lime is easily detected. Before the blowpipe it first blackens, and then becomes white, retaining however its form, and exibiting no appearance of fusion. Pulverised, it is readily dissolved in dilute muriatic acid; and if the excess of acid be not very considerable, the lime may be precipitated, in the form of an insoluble compound, by oxalat of ammonia.

The ammoniaco-magnesian phosphat, though seldom pure, may often be known by its whiteness, and its crystalline sparkling appearance. If a few particles of this salt (either as deposited in white sand in the urine, or detached from a calculus) be exposed to a gentle heat, or treated with a few drops of caustic potash, by either of these means a pungent smell of volatile alkali will be perceived. If the heat is urged by the blow-pipe, the phosphat of magnesia that remains becomes opake, and is capable of being imperfectly fused.

The fusible calculus readily melts under the flame of the blow-pipe, bubbles up and runs into a globule, pearly white, or perfectly transparent. It is also readily dissolved by acids.

Oxalat of lime is easily known by its appearance, at least in general. When heated, it swells and expands into a white efflorescence, which, when applied to paper stained with juice of violets, turns it green. This white alkaline substance is nothing but caustic lime deprived of the oxalic acid which was driven off by the heat. This variety of calculus is apt to decrepitate on the application of heat.

The cystic oxyd is easily known by its unstratified structure, peculiar colour, waxy appearance, and peculiar odour when heated. Its more ready solubility in acids and alkalis, in comparison with other calculi, will also distinguish it.

The compound calculi will not of course be so readily made out, but must be examined according to the rules just laid down, although the composition of these are with difficulty ascertained.

Chap. 7. *On some other Kinds of Animal Concretions not belonging to the urinary Passages, both in Man and other Animals.*

The author, in the present Chapter, makes some interesting observations; but, upon the whole, it does not appear to us that he has placed this division of his subject in the most interesting point of view. We find in it, that many of the secreting organs of the body will occasionally separate a quantity of carbonat or phosphat of lime; and also, that when magnesia, cheese, oat cake, &c. are taken in excess, concretions may be produced in the intestines, to the inconvenience of the patient.

We are also informed, that in a gentleman of philosophic temper, some curious appearances in the stools were ascertained, without the aid of *chemical analysis*, to proceed from the spawn of lobsters, which he admitted he had just before taken.

Comparative calculi from the stomach or intestines of various animals, are generally found to consist of the phosphat and carbonat of lime. Lithic acid, afforded but rarely in the excretions of animals, has been detected in large proportion in the excrements of the boa constricta, by Dr. Prout, a gentleman whose taste for chemical science has already led to several interesting results.

Chap. 8. *On the chemical and physiological Principles to be attended to in the Treatment of Calculous Disorders.*

We now come to the most important part of the Treatise, which the author commences by admitting what is invariably true, that the application of chemical principles will not enable us so to treat a patient, as to dissolve calculi already formed in the urinary passages.

The principles of treatment being connected with peculiarities in the secretion of urine, the state and composition of this fluid is very properly entered upon.

Some of the substances suspended in the urine are little soluble, and therefore more disposed to separate in a concrete form; these are phosphat of lime, phosphat of magnesia, and lithic acid.

Healthy urine is slightly acid, and reddens vegetable blues. This is owing to excess of phosphoric, and partly to the lactic and lithic acids, portions of each of these acids remaining disengaged or uncombined.

On standing, lithic acid and phosphat of lime spontaneously subside in the urine; but when putrefaction commences, ammonia being generated, unites with any uncombined acid, thereby occasioning a precipitation of the earthy and less soluble salts, especially the phosphats.

The author's remarks upon this part of his subject are highly interesting and exceedingly valuable. He observes, that if any alkali (a few drops of ammonia) be added to recent urine, a white cloud appears, and a sediment of the phosphats falls to the bottom. Lime-water produces a similar result, but in a more copious proportion.

On the addition of an acid, either phosphoric, muriatic, or acetic, the mixture being allowed to stand a few days, small reddish crystalline particles of lithic acid will be deposited on the sides of the vessel.

"It is on these two general facts that our principles of chemical treatment ultimately rest. Whenever the lithic secretion predominates, the alkalies are the appropriate remedies; and the acids, particularly the muriatic, are the agents to be resorted to when the calcareous or magnesian salts prevail in the deposit."

Dr. Marcet next explains, that with ease may alkalies be made to pass off in abundance by the kidnies, so as to effectually prevent excess of acid in the urine; but with regard to a similar use of acid medicines, the question is not easily determined. Mr. Brande states, that acids taken into the stomach are capable of being conveyed into the bladder. But although alkalies do, and acids may possibly reach the bladder, it is not possible by these means to make any material impression on a calculus already formed; we can, however, change the disposition of the system, so as to prevent the further growth of the concretion.

The administration of acid or alkali is easy, few stomachs being materially disturbed by moderate doses of either the mineral acids, or the carbonated alkalies. The author generally gives from five to twenty-five drops of muriatic acid in water, the dose being repeated several times a day.

The preparation most in use among the alkalies, is that called soda water, in a tumbler glass, of which a drachm of the carbonated alkali may be taken pleasantly.

The citric and carbonic acids are next taken up; and with relation to the latter of these bodies, the author dissents from Mr. Brande's opinion, that the carbonic acid is conveyed in excess into the urine, when taken medicinally. Dr. Marcet thinks such an opinion still highly improbable.

The use of magnesia is approved as an useful addition to the medical treatment of calculous disorders; but the author at the same time discriminates between the powers possessed by magnesia, and those which, in certain cases, render the alkalies decidedly superior to it.

The secretion of ropy mucous matter, which is frequently connected with these complaints, the Doctor thinks greatly assists in adding to the magnitude of the calculus; he also conceives that this is more particularly the case with concretions of the chalky or friable kind.

Alkalies tend to increase this inconvenience; whereas the muriatic acid, plentifully diluted, checks the excretion of mucus, in some instances very remarkably.

The oxalat of lime, the cystic oxyd, and the two new calculi described by the author, present difficulties as to the principle to be held in view in the treatment.

The mulberry calculus is totally incapable of being influenced by any acid reaching it through the system, while the cystic oxyd and xanthic calculus are soluble both in acids and alkalies. The fibrinous calculus is scarcely acted upon by any of the chemical re-agents.

A peculiar difficulty is, that no trace or vestige of any of these calculi are discoverable in the urine, consequently it is not easy to attempt the correcting the peculiar diathesis.

Upon the whole, the Doctor thinks, that where it is supposed the oxalat of lime is deposited, the exhibition of muriatic acid will have the best chance of doing good; and where cystic or xanthic deposits exist, the only mode is to adopt the good old rule, and try all things, holding fast that which is good.

The good effect of smart purgatives, in suspending the characteristics of the calculous diathesis, is particularly noticed, and certainly merits all the attention that can be paid to it, in a practical point of view.

The practice of injecting alkaline or acid solutions into the bladder, as proposed by Fourcroy, the author seems disposed to recommend, observing, that he by no means thinks the subject has yet been sufficiently investigated. Now upon this point we have some doubts, and are very sure that such inquiries ought (if made at all) to be conducted with extreme caution; for, independently of our own experience in this matter, we recollect that Mr. Howship, in his late observations on the diseases of the urinary organs, relates an interesting case, in which a contracted bladder, carefully injected with an ounce and a half of warm water only, became affected with a most severe attack of irritability, which did not leave the patient for weeks afterward.

The author, in conclusion, illustrates his subject by ten engravings, some of them coloured, in which he discriminates accurately the appearance peculiar to each kind of concretion.

As a view of the treatment most appropriate for these complaints, on chemical principles, the above work certainly stands alone, and superior to any thing we know of; but we conceive the author might still further improve it by entering a little more fully into the modes of analysing morbid urine, although what he has already accomplished deserves great praise.

